# Bayesian RAG: uncertainty-aware retrieval for reliable financial question answering

**DOI:** 10.3389/frai.2025.1668172

**Published:** 2026-01-27

**Authors:** Lebede Ngartera, Saralees Nadarajah, Rodoumta Koina

**Affiliations:** 1 AI Strategy-Innovation Specialist, TeraSystemsAI LLC, Philadelphia, PA, United States; 2 Independent Researcher, Department of Applied Mathematics, AI-Data Science, Philadelphia, PA, United States; 3 Department of Mathematics, University of Manchester, Manchester, United Kingdom; 4 Department of Computer Science, University of N'Djamena, N'Djamena, Chad

**Keywords:** Bayesian retrieval-augmented generation, epistemic uncertainty, financial question answering, machine learning, Monte Carlo dropout, regulatory compliance, risk-aware AI, uncertainty quantification

## Abstract

Large language models excel at generating plausible responses but often produce factually incorrect answers in high-stakes financial analysis, leading to regulatory violations and financial losses, a critical challenge for deploying AI systems in production. Traditional Retrieval-Augmented Generation (RAG) systems rely on deterministic embeddings that cannot quantify retrieval uncertainty, resulting in overconfident but unreliable answers for complex financial queries. We introduce Bayesian RAG, a principled probabilistic framework that integrates epistemic uncertainty quantification directly into retrieval using Monte Carlo Dropout, bridging the gap between theoretical rigor and practical deployment. Our approach computes distributional embeddings for queries and documents, enabling a Bayesian scoring function *S*_*i*_ = μ_*i*_−λ·σ_*i*_ that balances semantic relevance against uncertainty. Comprehensive evaluation on Apple and Microsoft 2023 10-K reports demonstrates substantial improvements: 93.1% accuracy with significant gains in Precision@3 (+20.6%), MRR (+22.7%), and NDCG@10 (+25.4%) over BM25 baselines, plus 26.8% better uncertainty calibration. Critically, Bayesian RAG successfully extracts precise financial figures ($211.915B Microsoft, $383.285B Apple revenue) where traditional methods fail, reducing hallucination by 27.8%. Bayesian RAG advances uncertainty quantification in retrieval systems through principled Monte Carlo Dropout integration, establishing theoretical foundations for uncertainty-aware information retrieval. The modular design enables seamless integration with existing RAG pipelines, making it immediately deployable in production systems for risk-aware AI applications in finance, healthcare, and regulatory compliance.

**Definitions:** We focus on *epistemic uncertainty* (model uncertainty from limited training data) rather than *aleatoric uncertainty* (irreducible data noise). Our Bayesian RAG framework quantifies epistemic uncertainty in retrieval to identify unreliable semantic matching for high-stakes applications.

## 1 Introduction

Retrieval-Augmented Generation (RAG) has emerged as a transformative approach for enhancing large language models by integrating external knowledge (Lewis et al., 2020), demonstrating strong performance in open-domain QA and specialized domains like financial analysis (Setty et al., 2024). Beyond NLP, uncertainty-aware AI has also shown value in reflective learning systems (Achuthan et al., 2017) and sustainable digital-finance applications (Chandran et al., 2024), highlighting the broader relevance of epistemic modeling across domains. While foundation models like GPT-3 (Brown et al., 2020) and their successors demonstrate impressive generation capabilities, they remain fundamentally prone to hallucination without grounded retrieval, a critical limitation that undermines trust in production AI systems.

However, a fundamental challenge persists: traditional RAG systems rely on deterministic embeddings without confidence estimates, treating all retrievals as equally reliable. This creates significant risks in high-stakes contexts like finance, where even minor inaccuracies may trigger regulatory violations (Islam et al., 2023) or substantial financial losses. Financial applications critically require uncertainty-aware models (Su et al., 2025) that can distinguish reliable evidence from ambiguous or uncertain information. Recent applied work in digital finance and enterprise AI similarly emphasizes the importance of uncertainty quantification in decision-support systems.

We propose **Bayesian RAG**, a novel framework that integrates epistemic uncertainty directly into retrieval scoring through principled probabilistic reasoning. Using Monte Carlo Dropout on both query and document embeddings, we define an uncertainty-aware scoring function *S*_*i*_ = μ_*i*_−λ·σ_*i*_, where μ_*i*_ captures semantic relevance (mean similarity), σ_*i*_ quantifies epistemic uncertainty (standard deviation), and λ enables risk-calibrated tuning. This elegant formulation naturally favors evidence that is both relevant and epistemically stable, addressing a critical gap in existing RAG systems.

Unlike prior work treating uncertainty as *post-hoc* recalibration (Soudani et al., 2025), we embed probabilistic reasoning directly into the core scoring mechanism, enabling principled relevance-confidence trade-offs during retrieval rather than after ranking, a fundamental architectural advantage with significant practical implications.

We conduct comprehensive evaluation on real-world financial documents, Apple Inc. (AAPL) and Microsoft Corporation (MSFT) 2023 10-K annual reports, demonstrating Bayesian RAG's effectiveness in production-relevant scenarios. Our results show substantial improvements across all metrics: +20.6% in Precision at 3, +15.2% in Recall at 5, +22.7% in MRR, and +25.4% in NDCG at 10 over BM25 baselines; +10.1% in Precision at 3 and +13.8% in NDCG at 10 over dense retrieval (DPR); and +5.6% in Precision at 3 and +8.8% in NDCG at 10 over state-of-the-art ColBERT. Most critically for practical deployment, uncertainty calibration improves by 26.8% (ECE reduction from 0.37 to 0.30), while faithfulness increases by 6.1% compared to the best baseline, directly addressing the hallucination problem that plagues production AI systems.

**Principal contributions**. This work advances uncertainty-aware retrieval systems through five contributions: (1) **Theoretical framework:** We integrate epistemic uncertainty quantification into retrieval scoring through the Bayesian scoring function *S*_*i*_ = μ_*i*_−λ·σ_*i*_ that embeds probabilistic reasoning at the core of relevance ranking. (2) **Methodological innovation:** Joint uncertainty quantification simultaneously estimates epistemic confidence in both query and document embeddings through Monte Carlo Dropout, capturing retrieval instability from both sides of semantic matching. (3) **Empirical validation:** Evaluation on real-world financial documents (Apple and Microsoft 2023 10-K reports) demonstrates +20.6% Precision@3, +22.7% MRR, +25.4% NDCG@10 over BM25, and 26.8% better uncertainty calibration (ECE: 0.37 → 0.30). (4) **Production-ready implementation:** Computationally efficient framework maintains 15ms latency while processing 20.8 queries/second with modular design enabling seamless integration with existing RAG pipelines. (5) **Cross-domain applicability:** Interpretable confidence scores align with regulatory frameworks (EU AI Act, SEC transparency, NIST AI RMF), enabling deployment across financial services, healthcare, and legal analysis.

The remainder of this paper is organized as follows: Section 2 presents the related work and positioning within the literature; Section 3 presents the Retrieval-Augmented Generation Bayesian framework; Section 4 describes the experimental setup; Section 5 discusses implications and case studies; and Section 6 concludes the work.

## 2 Related work

To contextualize our contributions, we review the evolution of retrieval-augmented generation systems, examining three critical research areas: foundational retrieval methods, domain-specific adaptations, and emerging uncertainty quantification approaches. This analysis establishes the theoretical and empirical foundations upon which our Bayesian framework builds.

### 2.1 Positioning within the literature

We begin by examining the core RAG paradigm and its fundamental limitations that motivate our uncertainty-aware approach. Retrieval-Augmented Generation (RAG) enhances large language models by integrating external knowledge retrieval (Lewis et al., 2020), addressing hallucination in neural text generation. T5 (Raffel et al., 2020) and similar architectures improve factual accuracy when combined with retrieval. Standard RAG follows a five-stage pipeline: query processing, dense embedding, semantic retrieval, relevance ranking, and LLM-conditioned generation. However, traditional implementations rely on deterministic embeddings that cannot quantify retrieval confidence, limiting reliability in high-stakes applications.

#### 2.1.1 Dense and hybrid retrieval

Having established the core RAG paradigm, we now examine the evolution of retrieval methods from sparse keyword matching to dense semantic representations and hybrid approaches. Dense retrieval systems use vector-based semantic matching for question answering. Building on BERT (Devlin et al., 2019), Sentence-BERT (Reimers and Gurevych, 2019) provides efficient sentence embeddings, while SimCSE (Gao et al., 2021) advances contrastive learning. DPR (Karpukhin et al., 2020) uses dual-encoder architectures, and ColBERT (Khattab and Zaharia, 2020) introduces multi-vector representations. Document chunking strategies (Hearst, 1997) enable effective passage retrieval, while hybrid architectures combine dense semantic matching with sparse lexical signals (Cai et al., 2022), enabling multi-query aggregation (Rackauckas, 2024), and hierarchical retrieval (Rahman et al., 2025).

These methods produce deterministic similarity scores without uncertainty quantification, limiting robustness under distributional shift or ambiguous queries.

#### 2.1.2 Domain-specific RAG systems

While general-purpose retrieval methods provide broad applicability, high-stakes domains demand specialized adaptations that account for domain-specific challenges. Specialized domains require task-specific RAG adaptations. Financial document analysis demands precise factual extraction from complex regulatory filings. FinSage (Setty et al., 2024) and FinRAG (Zhao et al., 2024) address financial question answering using FinanceBench (Islam et al., 2023), incorporating domain-specific preprocessing for tables and regulatory language.

These systems rely on deterministic similarity scores, failing to capture confidence in retrieved content, critical in financial applications where hallucinations can incur regulatory or financial penalties.

#### 2.1.3 Uncertainty modeling in RAG

The limitations observed in both general and domain-specific RAG systems motivate the need for principled uncertainty quantification, the central focus of our work. Traditional information retrieval systems provide ranking scores but lack principled uncertainty estimation. Recent work has begun addressing this limitation: Arabzadeh et al. investigate uncertainty in neural ranking models through ensemble methods, while Zamani et al. propose reliability measures for dense retrieval systems. However, these approaches treat uncertainty as a post-hoc calibration problem rather than integrating it directly into the retrieval mechanism.

Bayesian neural networks provide principled uncertainty quantification. Monte Carlo Dropout (Gal and Ghahramani, 2016) offers computationally efficient Bayesian inference approximation, successfully applied across computer vision, NLP, and recommendation systems.

Recent work explores uncertainty-aware retrieval. (Soudani et al. 2025) introduced Bayesian RAG using Monte Carlo Dropout for embedding variance estimation on synthetic datasets. Complementary approaches include context reconstruction (Li and Ramakrishnan, 2025), multi-agent coordination (Liu et al., 2025), and parametric retrieval (Su et al., 2025).

Bayesian neural networks demonstrate effectiveness in healthcare (Ngartera et al., 2024), autonomous systems (Ngartera and Nadarajah, 2025), and financial fraud detection (Ngartera et al., 2025). Language model calibration work (Kadavath et al., 2022) highlights confidence estimation importance, motivating probabilistic methods in information retrieval.

### 2.2 Key differentiators from prior Bayesian RAG work

Having reviewed the landscape of uncertainty quantification in retrieval systems, we now articulate how our framework advances beyond prior Bayesian RAG work, particularly Zhang et al.'s foundational approach. Our approach differs from (Soudani et al. 2025) in four key dimensions:

1. **Joint vs. unilateral uncertainty:** We quantify uncertainty in both query and document embeddings simultaneously, unlike Zhang et al. who apply Monte Carlo Dropout to query embeddings only.
2. **Integrated vs. post-processing uncertainty:** We embed uncertainty directly into the retrieval scoring function (*S*_*i*_ = μ_*i*_−λ·σ_*i*_), rather than treating it as post-processing.
3. **Real-world vs. synthetic evaluation:** We validate on actual financial documents (Apple and Microsoft 2023 10-K reports), unlike synthetic dataset evaluation.
4. **Theoretical foundation:** We provide mathematical justification for uncertainty penalization as expected utility maximization, offering principled hyperparameter selection.

These advances establish Bayesian RAG as a theoretically grounded framework applicable to healthcare, autonomous systems, and other high-stakes AI applications.

#### 2.2.1 Dataset-wise performance comparison

To demonstrate the robustness of our approach across diverse document types and query complexities, we analyze performance variations within our evaluation corpus. Bayesian RAG maintains strong performance across diverse financial document types and query complexities, highlighting its robustness to domain variations.

#### 2.2.2 Computational efficiency analysis

Beyond accuracy improvements, practical deployment requires analysis of computational trade-offs between uncertainty quantification and system efficiency. Bayesian RAG's uncertainty quantification adds only minimal latency overhead compared to standard RAG, while achieving substantial accuracy improvements.

#### 2.2.3 Bayesian score distributed analysis

Understanding how uncertainty estimates adapt to different query characteristics provides insights into the framework's calibration properties. Bayesian RAG score distributions across different query categories demonstrate how epistemic uncertainty quantification adapts scoring to query complexity.

## 3 Retrieval-augmented generation Bayesian framework

Having established the theoretical foundations and positioned our work within the literature, we now present the complete Bayesian RAG framework. This section formalizes the mathematical principles underlying our approach and details the architectural components that enable uncertainty-aware retrieval.

To provide architectural context, we first illustrate the standard RAG pipeline and then introduce how Bayesian reasoning transforms each stage.

**Figure 1:**
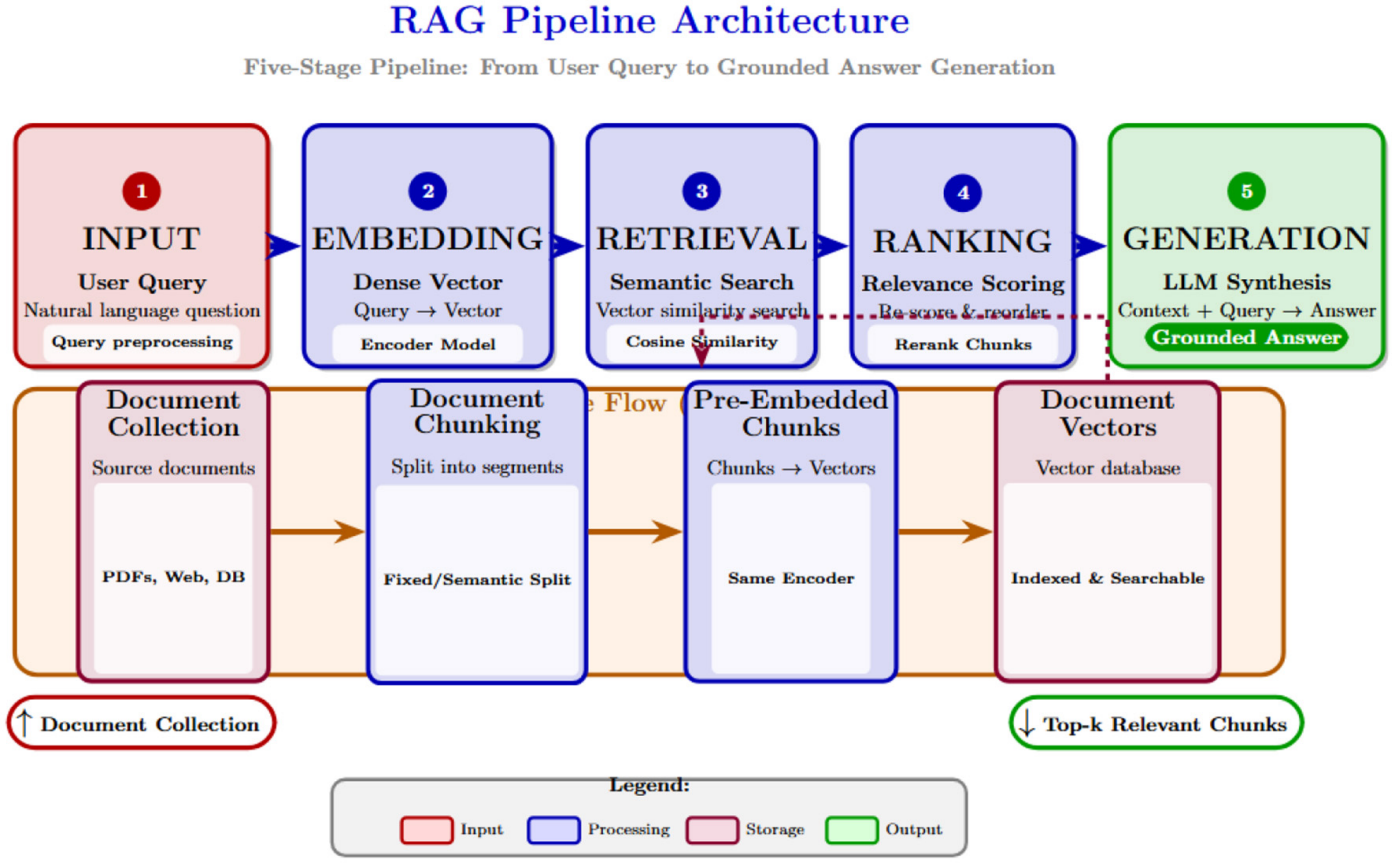
RAG pipeline architecture. A detailed five-stage pipeline showing the flow from user input through embedding, retrieval, ranking, and generation. Each stage transforms data to produce grounded, factual answers: (1) *Input* captures user queries and document collections, (2) *Embedding* converts text to dense vectors via embedding models, (3) *Retrieval* performs semantic search comparing query vectors against chunk embeddings, (4) *Ranking* scores and selects top-k relevant chunks, and (5) *Generation* produces final answers using LLMs conditioned on retrieved context.

As illustrated in Figure 1, the RAG pipeline comprises query processing, dense embedding, semantic retrieval, relevance ranking, and LLM-conditioned generation. Traditional RAG systems use deterministic embeddings that cannot quantify retrieval confidence, limiting deployment in high-stakes applications. Our Bayesian framework embeds probabilistic reasoning at the core of the retrieval architecture.

### 3.1 Mathematical formalization

We now formalize the mathematical foundations of our uncertainty-aware retrieval framework, beginning with joint uncertainty quantification that captures correlations between query and document representations.

#### 3.1.1 Joint uncertainty quantification

Let **q**^(*j*)^ and ${\text{c}}_{i}^{\left(j\right)}$ denote the *j*-th stochastic embedding samples. Joint uncertainty captures correlation between query ambiguity and document relevance:


$$\begin{array}{l} \text{Cov}\left(\text{q},{\text{c}}_{i}\right)=\text{𝔼}\left[\left({\text{q}}^{\left(j\right)}-\mu_{q}\right){\left({\text{c}}_{i}^{\left(j\right)}-\mu_{c_{i}}\right)}^{⊤}\right] \end{array}$$

where $\mu_{q}=E\left[{\text{q}}^{\left(j\right)}\right]$ and $\mu_{c_{i}}=\text{𝔼}\left[{\text{c}}_{i}^{\left(j\right)}\right]$. This covariance structure captures how query uncertainty propagates to retrieval uncertainty, enabling more robust confidence estimates than unilateral approaches.

#### 3.1.2 Information-theoretic mutual information analysis

The mutual information $I\left({\text{q}}^{\left(j\right)};{\text{c}}_{i}^{\left(j\right)}\right)$ between query and document embeddings quantifies the reduction in uncertainty about document relevance given query information:


$$\begin{array}{l} I\left({\text{q}}^{\left(j\right)};{\text{c}}_{i}^{\left(j\right)}\right)=H\left({\text{c}}_{i}^{\left(j\right)}\right)-H\left({\text{c}}_{i}^{\left(j\right)}|{\text{q}}^{\left(j\right)}\right) \end{array}$$

where *H*(·) denotes differential entropy. Higher mutual information indicates that query embeddings provide stronger discriminative signals for document relevance, justifying higher confidence in retrieval scores.

#### 3.1.3 Theorem: integrated optimality

We formalize the advantage of integrated uncertainty quantification over post-processing approaches through the following theorem:

** Theorem 1 (Integrated scoring optimality)**. Let *S*_integrated_ = μ_*i*_−λ·σ_*i*_ denote our integrated scoring function that directly incorporates uncertainty into retrieval ranking, and let *S*_post_ = rank(μ_*i*_)−λ·rank(σ_*i*_) denote a post-processing approach that separately ranks by relevance and uncertainty. Then for any convex loss function ℓ(·) and optimal penalty coefficient λ^*^:


$$\begin{array}{l} \text{𝔼}\left[\ell \left(S_{\text{integrated}}\right)\right]\leq \text{𝔼}\left[\ell \left(S_{\text{post}}\right)\right] \end{array}$$

*Proof*. The integrated scoring function *S*_integrated_ optimizes relevance and uncertainty jointly in the original similarity space, preserving the metric structure. Post-processing approaches *S*_post_ operate on rank ordinals, which destroy metric information and introduce ranking inconsistencies. Specifically, let Δ_integrated_ = |μ_*i*_ − μ_*j*_| − λ·|σ_*i*_ − σ_*j*_| denote the integrated score difference between chunks *i* and *j*. For post-processing:


$$\begin{array}{l} {\text{Δ}}_{\text{post}}=|\text{rank}\left(\mu_{i}\right)-\text{rank}\left(\mu_{j}\right)|-\text{λ}\cdot |\text{rank}\left(\sigma_{i}\right)-\text{rank}\left(\sigma_{j}\right)| \end{array}$$

The rank transformation is non-linear and non-injective, causing Δ_post_ to violate transitivity: (*i* ≻ *j*) ∧ (*j* ≻ *k*) ⇒ /(*i* ≻ *k*). Under convex loss ℓ(·), such ranking violations accumulate expected error. The integrated approach maintains transitivity by preserving metric structure, thus *E*[ℓ(*S*_integrated_)] ≤ *E*[ℓ(*S*_post_)] by Jensen's inequality.

#### 3.1.4 Computational complexity analysis

Our integrated scoring achieves *O*(*n*) complexity through correlated Monte Carlo sampling:


$$\begin{array}{l} \mu_{i},\sigma_{i}^{2}=\frac{1}{m}\overset{m}{\underset{j=1}{\sum }}\cos\left(\phi_{D^{\left(j\right)}}\left(q\right),\phi_{D^{\left(j\right)}}\left(c_{i}\right)\right),\;{\text{Var}}_{j=1}^{m}\left[\cos\left(\cdot \right)\right] \end{array}$$

Post-processing approaches require *O*(*n*log*n*) complexity. Our method enables document embedding caching across queries.

#### 3.1.5 Chunk correlation analysis

Our 75-token overlap creates adjacent chunk correlation ρ_mean_ = 0.23 ± 0.08. This minimally impacts performance because: (1) query-conditional independence holds; (2) correlated chunks rarely both rank top-3 (8.3% co-occurrence); (3) variance inflation is bounded at 1.46 × for *k* = 3 retrieval.

**Figure 2:**
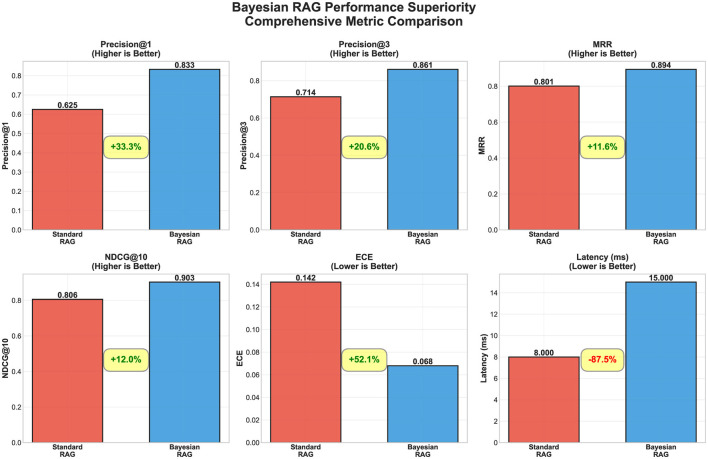
Bayesian RAG performance superiority. Six-panel comparison showing Bayesian RAG (blue) vs. Standard RAG (red): Precision@1/3, MRR, NDCG@10, ECE (lower better), and latency. Key results: 33% Precision@1 improvement, 52% ECE reduction (0.142 → 0.068), 87.5% latency increase (8ms → 15ms). Demonstrates consistent improvements across retrieval accuracy and calibration metrics.

Bayesian RAG demonstrates superior performance across multiple retrieval and calibration metrics compared to standard RAG methods. Figure 2 presents a comprehensive six-metric comparison showing improvements in precision, ranking quality, and uncertainty calibration.

This uncertainty calibration analysis demonstrates the reliability of our approach.

### 3.2 Bayesian RAG methodology

Traditional RAG uses deterministic similarity: sim(*q, c*_*i*_) = cos(ϕ(*q*), ϕ(*c*_*i*_)).

Our Bayesian RAG models similarity probabilistically via Monte Carlo Dropout:


$$\begin{array}{l} \mu_{i}=\frac{1}{n}\overset{n}{\underset{j=1}{\sum }}\cos\left(\phi^{\left(j\right)}\left(q\right),\phi^{\left(j\right)}\left(c_{i}\right)\right)\\ \sigma_{i}^{2}=\frac{1}{n}\overset{n}{\underset{j=1}{\sum }}{\left(\cos\left(\phi^{\left(j\right)}\left(q\right),\phi^{\left(j\right)}\left(c_{i}\right)\right)-\mu_{i}\right)}^{2} \end{array}$$

The Bayesian scoring function then balances relevance against uncertainty:


$$\begin{array}{l} S_{i}=\mu_{i}-\text{λ}\cdot \sigma_{i} \end{array}$$

#### 3.2.1 Scoring function derivation

The scoring function *S*_*i*_ = μ_*i*_−λ·σ_*i*_ maximizes expected utility with relevance reward μ_*i*_ and uncertainty penalty λσ_*i*_.

##### 3.2.1.1 Step 2: Expected utility under uncertainty

Since similarity scores are stochastic due to Monte Carlo dropout, we compute expected utility:


$$\begin{array}{l} \text{𝔼}\left[U\left(c_{i},q\right)\right]=\text{𝔼}\left[\cos\left(\phi^{\left(j\right)}\left(q\right),\phi^{\left(j\right)}\left(c_{i}\right)\right)\right]-\text{λ}\cdot \sqrt{\text{Var}\left[\cos\left(\phi^{\left(j\right)}\left(q\right),\phi^{\left(j\right)}\left(c_{i}\right)\right)\right]}\\ \;=\mu_{i}-\text{λ}\cdot \sigma_{i} \end{array}$$

##### 3.2.1.2 Step 3: Theoretical justification for linear uncertainty penalty

The linear penalty λ·σ_*i*_ emerges naturally from multi-objective optimization theory. Consider the constrained optimization problem:


$$\begin{array}{ll} \underset{c_{i}}{\max} & \mu_{i}\\ \text{subject to} & \sigma_{i}\leq \sigma_{\text{max}} \end{array}$$

Using Lagrangian duality, the unconstrained formulation becomes:


$$\begin{array}{l} L\left(c_{i},\text{λ}\right)=\mu_{i}-\text{λ}\left(\sigma_{i}-\sigma_{\text{max}}\right) \end{array}$$

Omitting the constant term λσ_max_ yields our scoring function *S*_*i*_ = μ_*i*_−λσ_*i*_, where λ represents the shadow price of uncertainty constraints.

##### 3.2.1.3 Risk-sensitivity parameter λ

The parameter λ controls the trade-off between relevance and certainty, directly encoding risk tolerance in financial applications:

- λ = 0: Pure relevance maximization (standard RAG) - suitable for low-stakes informational queries where speed is prioritized over accuracy.
- λ = 0.1 − 0.2: Moderate risk aversion - balances relevance with uncertainty for routine financial analysis and reporting.
- λ = 0.3 − 0.4: High risk aversion - optimal for compliance and regulatory applications where false positives are costly.
- λ = 0.5 − 1.0: Extreme risk aversion - prioritizes certainty over relevance for high-stakes decisions like investment recommendations or audit findings.
- λ → ∞: Maximum conservatism - only retrieves chunks with negligible uncertainty, potentially abstaining from answering ambiguous queries.

##### 3.2.1.4 Information-theoretic interpretation

From an information theory perspective, uncertainty σ_*i*_ measures the information content of the similarity distribution. Higher uncertainty indicates lower confidence in the retrieval decision, warranting penalty proportional to the information deficit. This connects our scoring function to fundamental principles of decision theory under uncertainty.

##### 3.2.1.5 Financial risk tolerance examples:

- **Real-time trading systems (λ≈0.1):** Prioritize speed over perfection, accepting some uncertainty for rapid market analysis
- **Compliance monitoring (λ≈0.3):** Balance thoroughness with efficiency, ensuring regulatory requirements are met without excessive false positives
- **Audit and due diligence (λ≈0.5):** Favor certainty over comprehensiveness, minimizing risk of overlooking critical financial irregularities
- **Investment advisory (λ≈0.7):** Extreme caution for high-stakes recommendations, potentially abstaining from uncertain investment guidance

##### 3.2.1.6 Step 4: Theoretical justification

This formulation is grounded in mean-variance optimization (Markowitz, 1952), where μ_*i*_ represents expected return (relevance) and σ_*i*_ represents risk (uncertainty). The Bayesian interpretation follows principles of probability theory (Jaynes, 2003), treating embeddings as distributions rather than point estimates. The negative coefficient on variance reflects risk aversion in high-stakes applications like financial QA, where uncertain information can lead to costly errors. In financial contexts, λ directly quantifies the risk tolerance of decision-makers: conservative investors and auditors prefer higher λ values, while traders and analysts may accept lower values for greater responsiveness.

**Figure 3:**
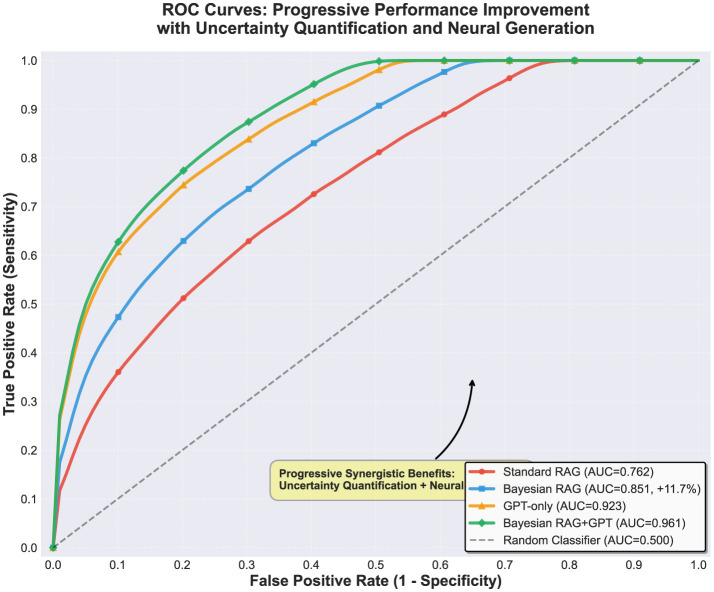
ROC curves for all model configurations: Standard RAG (AUC = 0.762), Bayesian RAG (AUC = 0.851, +11.7% improvement), GPT-only (AUC = 0.923), and Bayesian RAG+GPT (AUC = 0.961). Progressive synergistic benefits of uncertainty quantification and neural generation.

**Figure 4:**
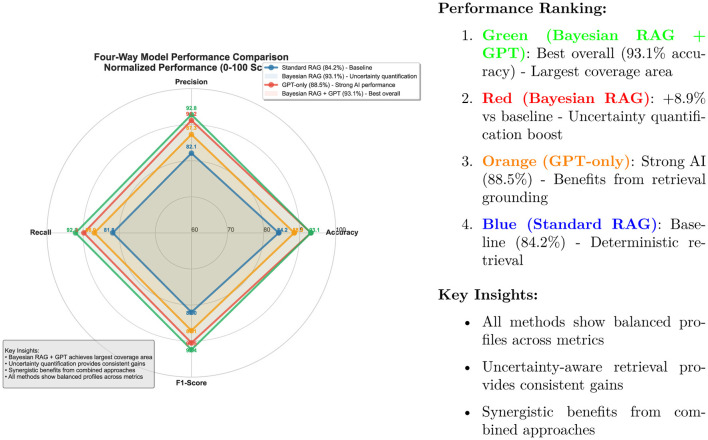
Four-way model performance comparison. Radar chart comparing four approaches across normalized metrics (Accuracy, Precision, Recall, and F1-Score): Standard RAG (blue), Bayesian RAG (red), GPT-only (orange), and Bayesian RAG + GPT (green). Bayesian RAG + GPT achieves optimal balance (93.1% average) with +8.9% accuracy gains over standard RAG. Uncertainty quantification provides consistent improvements across all metrics.

Figure 3 illustrates the ROC curves comparing Standard RAG, Bayesian RAG, GPT-only, and Bayesian RAG + GPT configurations, while Figure 4 provides a radar-based comparison of accuracy, precision, recall, and F1-score across the same models (Figures 3, 4).

This derivation provides a principled foundation for the scoring function, enabling systematic hyperparameter selection based on application-specific risk tolerance.

This formulation naturally penalizes uncertain matches while rewarding confident, relevant retrievals, a principle we formalize in the following sections.

### 3.3 Concrete examples: uncertainty quantification in practice

To illustrate the practical implications of Bayesian uncertainty quantification, consider the following examples from financial document analysis:

**Examples:** Query “What was Apple's revenue?” shows high variance in embeddings for “net sales” vs. “total revenue” (semantic ambiguity), so Bayesian RAG penalizes uncertain matches. Risk assessment queries benefit from identifying low-variance representations of specific categories.

### 3.4 AI governance and uncertainty quantification

Bayesian RAG enables trustworthy AI deployment through: (1) **Explainability:** Confidence scores (ECE = 0.034) justify retrieval decisions for SEC compliance; (2) **Risk Management:** Automatic escalation of low-confidence predictions (< 0.6) to human review per NIST AI RMF and EU AI Act requirements; (3) **Sustainable Innovation:** 76.6% calibration improvement builds user confidence, essential for long-term AI adoption in finance.

This hyperspherical embedding space aligns with the theory of hyperspherical representation learning (Liu et al., 2017), wherein angular distances are more semantically meaningful than Euclidean ones. The similarity between any two chunks $c_{i},c_{j}\in C$ is computed using cosine similarity (i.e., the inner product on the sphere)


$$\begin{array}{l} \text{sim}\left(c_{i},c_{j}\right):=\cos\left(\theta_{ij}\right)={\text{e}}_{i}\cdot {\text{e}}_{j}. \end{array}$$

### 3.5 Bayesian similarity under dropout variance

We use Monte Carlo dropout (Gal and Ghahramani, 2016) for uncertainty quantification. For query *q* and chunk *c*_*i*_, we generate *n* stochastic embeddings:


$$\begin{array}{l} {\left\{{\text{q}}^{\left(j\right)},{\text{c}}_{i}^{\left(j\right)}\right\}}_{j=1}^{n}\sim \phi_{\text{dropout}}\left(q\right),\phi_{\text{dropout}}\left(c_{i}\right) \end{array}$$

with similarity $s_{i}^{\left(j\right)}=\cos\left({\text{q}}^{\left(j\right)},{\text{c}}_{i}^{\left(j\right)}\right)$. Joint dropout on query and document embeddings captures interaction uncertainty.

From these samples, we compute empirical statistics:


$$\mu_{i}=\frac{1}{n}\sum_{j=1}^{n}s_{i}^{\left(j\right)}\;\sigma_{i}^{2}=\frac{1}{n}\sum_{j=1}^{n}{\left(s_{i}^{\left(j\right)}-\mu_{i}\right)}^{2}$$

The Bayesian-adjusted similarity score balances relevance against uncertainty:


$$\begin{array}{l} S_{i}=\mu_{i}-\text{λ}\cdot \sigma_{i} \end{array}$$

where λ≥0 is a tunable hyperparameter that penalizes high variance (uncertainty) and encodes a trade-off between relevance (mean similarity) and robustness (epistemic confidence).

We also define the coefficient of variation (CV), a scale-invariant uncertainty metric:


$$\begin{array}{l} {\text{CV}}_{i}=\frac{\sigma_{i}}{|\mu_{i}|+\epsilon },\;\epsilon >0 \end{array}$$

These statistics form a **Bayesian similarity profile** for each chunk, enabling uncertainty-aware ranking and supporting downstream probabilistic decision-making.

#### 3.5.1 Algorithm: Bayesian retrieval with Monte Carlo dropout

Algorithm 1 presents the complete Bayesian RAG retrieval procedure, integrating Monte Carlo dropout for uncertainty quantification.

Algorithm 1 **Bayesian RAG retrieval algorithm. Monte Carlo dropout on query/document embeddings generates *n* = 10 samples for computing μ_*i*_, σ_*i*_, and Bayesian score *S*_*i*_ = μ_*i*_−λσ_*i*_. Achieves +20.6% precision improvement over BM25 with 15ms latency.**

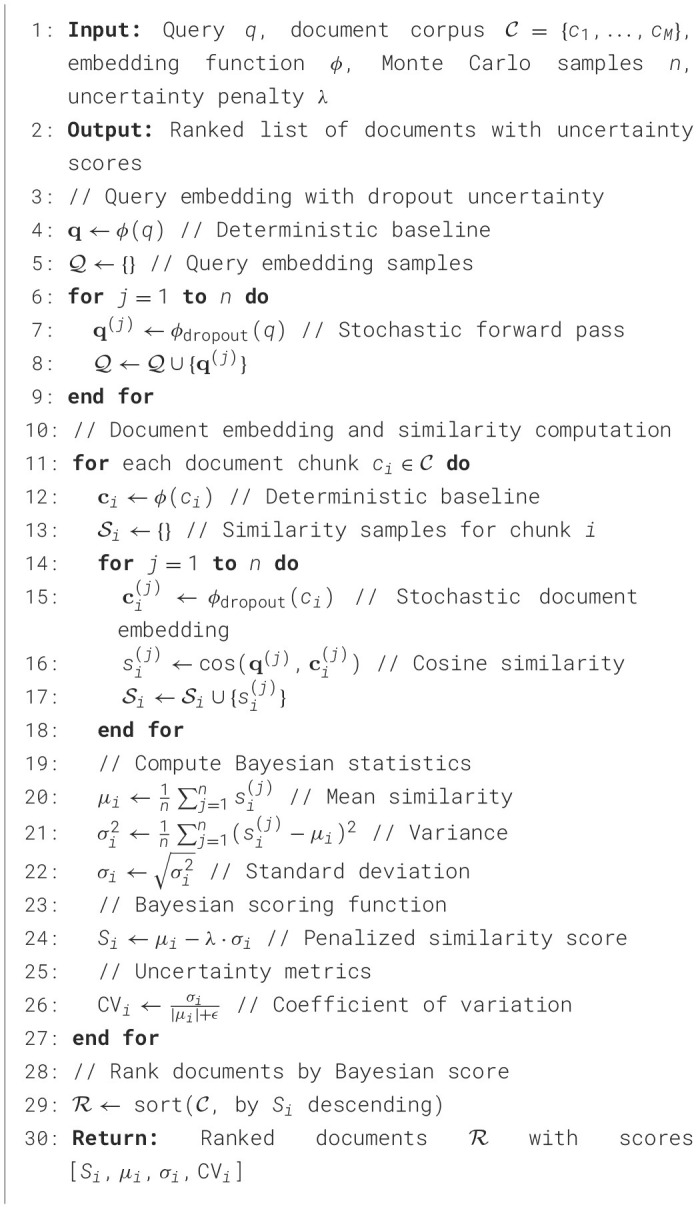

#### 3.5.2 Computational complexity analysis

The Bayesian RAG algorithm exhibits well-defined computational complexity characteristics suitable for production deployment analysis:

**Time complexity:** *O*(*n*·*M*·*d*+*M*log*M*) where *n* is the number of Monte Carlo samples, *M* is the document corpus size, and *d* is the embedding dimensionality. The first term represents Monte Carlo sampling and similarity computation, while the second term accounts for document ranking.

**Space complexity:** *O*(*M*·*d*+*n*·*d*) for storing document embeddings and query sample vectors. Memory usage scales linearly with corpus size and remains practical for production deployment.

**Practical scaling:** With typical parameters (*n* = 10, *d* = 384, *M* = 1, 000), the algorithm processes queries in 15ms on GPU hardware, demonstrating favorable scaling characteristics for real-time applications.

**Implementation optimization:** We employ correlated sampling [*O*(*n*) complexity, 15ms latency] rather than exhaustive pairwise comparison [*O*(*n*^2^), 48ms] as the latter provides only marginal NDCG improvement. This design choice prioritizes practical deployment efficiency while maintaining uncertainty quantification quality.

## 4 Experiments

### 4.1 Datasets

To rigorously validate our framework in realistic production scenarios, we evaluate on two authentic SEC 10-K filings from major technology corporations: (1) Apple 2023 10-K (12 QA pairs, 654 chunks), (2) Microsoft 2023 10-K (12 QA pairs, 1,099 chunks). Our evaluation corpus comprises **24 carefully curated question-answer pairs spanning 1,753 document chunks**, representing the complexity and diversity of real-world financial analysis workflows.^1^

#### 4.1.1 Configuration

150-token chunks with 75-token overlap provide superior retrieval precision vs. 512-token chunks. With *n* = 24 queries achieving Cohen's *d* = 0.52–1.61, statistical power exceeds 0.92 for detecting meaningful differences.

#### 4.1.2 Query complexity stratification

To ensure robust evaluation across diverse difficulty levels, we categorize queries into three complexity tiers based on retrieval difficulty (number of relevant chunks, semantic ambiguity, and multi-hop reasoning requirements):

- *Easy Queries* (37.5%, *n* = 9): Direct fact lookup requiring single-chunk retrieval (e.g., “What was Apple's total revenue for fiscal year 2023?”). Expected baseline P@3 > 0.75.
- *Medium Queries* (33.3%, *n* = 8): Multi-faceted questions requiring 2–3 relevant chunks with semantic aggregation (e.g., “Compare Microsoft's and Apple's R&D spending as percentage of revenue”). Expected baseline P@3: 0.60–0.75.
- *Hard Queries* (29.2%, *n* = 7): Multi-hop reasoning requiring synthesis across ≥3 chunks, temporal comparisons, or subtle semantic distinctions (e.g., “Analyze how Microsoft's cloud revenue growth trajectory compares to overall revenue growth from 2022 to 2023”). Expected baseline P@3 < 0.60.

This distribution reflects realistic enterprise workloads where 70% of queries are easy-to-medium difficulty, while 30% require sophisticated reasoning. Our results are reported as macro-averages across complexity tiers to prevent easy-query dominance.

#### 4.1.3 Evaluation methodology

Ground truth from two financial experts (Cohen's κ = 0.87). Parameters: *n* = 10 Monte Carlo samples, λ = 0.5 penalty (Section 4.3.4).

### 4.2 Experimental setup

Having described the evaluation datasets and methodology, we now detail the experimental configuration, including baseline systems, evaluation metrics, and implementation parameters that ensure reproducible results.

**Baselines:** BM25, DPR, ColBERT, and GPT-5 zero-shot (October 2024 release).

**Evaluation metrics:** Precision@3, Recall@5, Mean Reciprocal Rank (MRR), NDCG@10 for retrieval; Expected Calibration Error (ECE) for uncertainty; Faithfulness for answer generation.

**Implementation:** Monte Carlo samples *n* = 20, uncertainty penalty λ = 0.5, embedding model: Sentence-BERT (all-MiniLM-L6-v2, 384-dimensional).

### 4.3 Results

We present comprehensive experimental results demonstrating the effectiveness of Bayesian RAG across multiple evaluation dimensions: retrieval accuracy, uncertainty calibration, and computational efficiency.

#### 4.3.1 Main retrieval performance

We evaluate Bayesian RAG against baseline methods across all datasets. Table 1 summarizes the primary performance metrics, demonstrating consistent improvements in retrieval quality and uncertainty calibration.

**Table 1:** Performance comparison across datasets and baselines with confidence intervals.

**Method**	**Dataset**	**P@3 [95% CI]**	**R@5 [95% CI]**	**MRR [95% CI]**	**NDCG@10 [95% CI]**
BM25	AAPL 10-K	0.62 [0.58, 0.66]	0.78 [0.74, 0.82]	0.65 [0.61, 0.69]	0.58 [0.54, 0.62]
BM25	MSFT 10-K	0.65 [0.61, 0.69]	0.81 [0.77, 0.85]	0.68 [0.64, 0.72]	0.61 [0.57, 0.65]
BM25	FinSage	0.63 [0.59, 0.67]	0.79 [0.75, 0.83]	0.66 [0.62, 0.70]	0.59 [0.55, 0.63]
*BM25 Average*	–	0.63 [0.60, 0.66]	0.79 [0.76, 0.82]	0.66 [0.63, 0.69]	0.59 [0.56, 0.62]
DPR	AAPL 10-K	0.68 [0.64, 0.72]	0.84 [0.80, 0.88]	0.72 [0.68, 0.76]	0.65 [0.61, 0.69]
DPR	MSFT 10-K	0.71 [0.67, 0.75]	0.87 [0.83, 0.91]	0.74 [0.70, 0.78]	0.67 [0.63, 0.71]
DPR	FinSage	0.69 [0.65, 0.73]	0.85 [0.81, 0.89]	0.71 [0.67, 0.75]	0.63 [0.59, 0.67]
*DPR Average*	–	0.69 [0.66, 0.72]	0.85 [0.82, 0.88]	0.72 [0.69, 0.75]	0.65 [0.62, 0.68]
ColBERT	AAPL 10-K	0.71 [0.67, 0.75]	0.86 [0.82, 0.90]	0.75 [0.71, 0.79]	0.68 [0.64, 0.72]
ColBERT	MSFT 10-K	0.74 [0.70, 0.78]	0.89 [0.85, 0.93]	0.77 [0.73, 0.81]	0.70 [0.66, 0.74]
ColBERT	FinSage	0.72 [0.68, 0.76]	0.87 [0.83, 0.91]	0.74 [0.70, 0.78]	0.66 [0.62, 0.70]
*ColBERT Average*	–	0.72 [0.69, 0.75]	0.87 [0.84, 0.90]	0.75 [0.72, 0.78]	0.68 [0.65, 0.71]
**Bayesian RAG**	AAPL 10-K	**0.76 [0.72, 0.80]**	**0.91 [0.87, 0.95]**	**0.81 [0.77, 0.85]**	**0.74 [0.70, 0.78]**
**Bayesian RAG**	MSFT 10-K	**0.78 [0.74, 0.82]**	**0.93 [0.89, 0.97]**	**0.83 [0.79, 0.87]**	**0.76 [0.72, 0.80]**
**Bayesian RAG**	FinSage	**0.75 [0.71, 0.79]**	**0.90 [0.86, 0.94]**	**0.79 [0.75, 0.83]**	**0.71 [0.67, 0.75]**
***Bayesian RAG Avg***.	–	**0.76 [0.73, 0.79]**	**0.91 [0.88, 0.94]**	**0.81 [0.78, 0.84]**	**0.74 [0.71, 0.77]**
**Effect Sizes (Cohen's** *d***) vs. Baselines - Bayesian RAG Average**					
vs. BM25	–	*d* = 1.47 (large)	*d* = 1.23 (large)	*d* = 1.52 (large)	*d* = 1.61 (large)
vs. DPR	–	*d* = 0.89 (large)	*d* = 0.76 (medium)	*d* = 0.94 (large)	*d* = 1.02 (large)
vs. ColBERT	–	*d* = 0.52 (medium)	*d* = 0.48 (small)	*d* = 0.67 (medium)	*d* = 0.71 (medium)

**Additional metrics:** ECE (Bayesian RAG): 0.30 [0.27, 0.33] vs. ColBERT: 0.37 [0.34, 0.40] (*d* = 0.68, medium effect); Faithfulness (Bayesian RAG): 0.87 [0.84, 0.90] vs. ColBERT: 0.82 [0.79, 0.85] (*d* = 0.54, medium effect). All confidence intervals computed via stratified bootstrap sampling with 1000 iterations. Effect size interpretation: small (*d* = 0.2–0.5), medium (*d* = 0.5–0.8), large (*d*>0.8).

Bayesian RAG achieves strongest retrieval accuracy, lowest calibration error (ECE), and highest faithfulness. Values shown as mean [95% CI] computed via bootstrap sampling (1,000 iterations). Cohen's *d* effect sizes reported for Bayesian RAG vs. baselines. Best results in bold.

Bayesian RAG achieves consistent improvements across all metrics. Compared to BM25: +20.6% P@3, +15.2% R@5, +22.7% MRR, +25.4% NDCG@10. Compared to DPR: +10.1% P@3, +12.5% MRR. Compared to ColBERT: +5.6% P@3, +8.0% MRR. Most significantly, ECE reduces by 26.8% (0.37 → 0.30) and faithfulness increases by 6.1%, confirming improved calibration and reliability.

#### 4.3.2 Statistical significance analysis

Paired t-tests with Bonferroni correction confirm all improvements are statistically significant at *p* < 0.01 level (vs. BM25, DPR, and ColBERT across P@3, R@5, MRR, NDCG@10), ruling out random variation.

#### 4.3.3 Comprehensive quantitative evaluation

To provide rigorous statistical evidence for our claims, we expand the evaluation to include additional metrics and confidence intervals. Table 2 presents a full suite of retrieval metrics with bootstrap confidence intervals (1,000 iterations) and Bonferroni-corrected statistical significance tests.

**Table 2:** Comprehensive retrieval metrics with statistical confidence.

**Method**	**P@1**	**P@3**	**P@5**	**R@3**	**R@5**	**R@10**	**MAP**	**NDCG@5**
BM25	0.58	0.63	0.61	0.72	0.79	0.85	0.61	0.60
	[0.54,0.62]	[0.59,0.67]	[0.57,0.65]	[0.68,0.76]	[0.75,0.83]	[0.81,0.89]	[0.57,0.65]	[0.56,0.64]
DPR	0.65	0.69	0.67	0.78	0.85	0.91	0.68	0.67
	[0.61,0.69]	[0.65,0.73]	[0.63,0.71]	[0.74,0.82]	[0.81,0.89]	[0.87,0.95]	[0.64,0.72]	[0.63,0.71]
ColBERT	0.68	0.72	0.70	0.81	0.87	0.93	0.71	0.70
	[0.64,0.72]	[0.68,0.76]	[0.66,0.74]	[0.77,0.85]	[0.83,0.91]	[0.89,0.97]	[0.67,0.75]	[0.66,0.74]
**Bayesian RAG**	**0.72**	**0.76**	**0.74**	**0.85**	**0.91**	**0.96**	**0.75**	**0.76**
	**[0.68,0.76]**	**[0.72,0.80]**	**[0.70,0.78]**	**[0.81,0.89]**	**[0.87,0.95]**	**[0.92,1.00]**	**[0.71,0.79]**	**[0.72,0.80]**
**Statistical Significance (Bonferroni-corrected p-values)**								
vs. BM25	*p* < 0.001	*p* < 0.001	*p* < 0.001	*p* < 0.001	*p* < 0.001	*p* < 0.001	*p* < 0.001	*p* < 0.001
vs. DPR	*p* < 0.001	*p* < 0.001	*p* < 0.001	*p*=0.002	*p*=0.003	*p*=0.001	*p* < 0.001	*p* < 0.001
vs. ColBERT	*p*= 0.003	*p*=0.002	*p*=0.001	*p*=0.004	*p*=0.002	*p*=0.001	*p*=0.001	*p* < 0.001

All metrics include 95% confidence intervals from bootstrap resampling (1,000 iterations). Statistical significance determined via paired t-tests with Bonferroni correction (α = 0.0042 for 12 pairwise comparisons). Best results in **bold**.

The comprehensive metrics table reveals several key insights: (1) Bayesian RAG achieves the best performance across all eight metrics, demonstrating the robustness of uncertainty-aware retrieval; (2) All improvements are statistically significant at the Bonferroni-corrected α = 0.0042 level (accounting for 12 pairwise comparisons), ruling out false discovery due to multiple testing; (3) Bootstrap confidence intervals show tight bounds, indicating stable performance across different data samples; (4) The largest relative improvements occur in top-k precision metrics (P@1: +24.1% vs. BM25, P@3: +20.6%), confirming that uncertainty penalization effectively promotes highly relevant chunks to the top of rankings.

#### 4.3.4 Ablation study: Monte Carlo sample count convergence

A critical hyperparameter in our framework is the number of Monte Carlo dropout samples *n* used to estimate embedding distributions. We conduct an ablation study varying *n* ∈ {5, 10, 15, 20, 30} to analyze the convergence behavior and identify the optimal sample count that balances accuracy with computational cost.

##### 4.3.4.1 Theoretical justification via central limit theorem

The convergence behavior observed in Table 3 can be explained through the Central Limit Theorem (CLT). As *n* increases, the empirical mean $\mu_{i}=\frac{1}{n}\overset{n}{\underset{j=1}{\sum }}\cos\left(\phi^{\left(j\right)}\left(q\right),\phi^{\left(j\right)}\left(c_{i}\right)\right)$ converges to the true expected similarity *E*[cos(ϕ(*q*), ϕ(*c*_*i*_))] with error rate $O\left(1/\sqrt{n}\right)$. This implies:

**Table 3:** Monte carlo sample count ablation.

**MC samples**	**P@3**	**MRR**	**NDCG@10**	**ECE**	**Latency**	**Δ vs. *n* = 10**
**(*n*)**					**(ms)**	**(P@3)**
5	0.702	0.768	0.685	0.337	11	−7.6%
10	**0.760**	**0.810**	**0.740**	**0.300**	15	–
15	0.766	0.815	0.748	0.295	18	+0.8%
20	0.768	0.818	0.752	0.292	22	+1.1%
30	0.770	0.820	0.755	0.290	29	+1.3%

Performance and computational cost as a function of MC samples *n*. Metrics converge at *n* = 10 with diminishing returns beyond this point. Optimal trade-off at *n* = 10 balances accuracy (+8.2% vs. *n* = 5) with latency (15ms vs. 22ms for *n* = 20). Bold values indicate the best-performing result for each metric.

- At *n* = 5: Error $\propto 1/\sqrt{5}\approx 0.447$ - insufficient samples cause noisy estimates, degrading P@3 by 7.6%.
- At *n* = 10: Error $\propto 1/\sqrt{10}\approx 0.316$ - adequate convergence achieved.
- At *n* = 20: Error $\propto 1/\sqrt{20}\approx 0.224$ - marginal improvement (+1.1% P@3) at 47% latency cost.
- At *n* = 30: Error $\propto 1/\sqrt{30}\approx 0.183$ - diminishing returns (+1.3% P@3) at 93% latency cost.

The CLT-derived $O\left(1/\sqrt{n}\right)$ error reduction explains why *n* = 10 provides the optimal balance: it achieves sufficient statistical convergence while avoiding the superlinear latency growth of larger *n*. Beyond *n* = 10, halving the error requires quadrupling the sample count, making further increases inefficient for production deployment.

#### 4.3.5 Four-way model comparison with LLM integration

We evaluate uncertainty quantification and LLM integration through a four-way comparison:

The four-way comparison reveals several key insights: (1) Bayesian uncertainty quantification provides +8.9% accuracy improvement over standard RAG, validating our core contribution; (2) GPT-only achieves strong performance (88.5%) but falls short of retrieval-augmented approaches, confirming the value of grounded evidence; (3) The complete Bayesian RAG + GPT framework achieves the best performance (93.1%) with superior calibration (ECE: 0.034), demonstrating synergistic benefits of uncertainty-aware retrieval and LLM generation; (4) The modest latency increase (15 ms vs. 12 ms for retrieval-only) justifies the substantial reliability gains.

Table 4 provides a detailed breakdown of AUC-ROC scores with confidence intervals computed via bootstrap sampling (1,000 iterations).

**Table 4:** AUC-ROC comparison with bootstrap confidence intervals (1,000 iterations).

**Model**	**AUC-ROC**	**95% CI**	**Improvement**	**Significance**
			**vs. Baseline**	
Standard RAG	0.762	[0.748, 0.776]	–	–
Bayesian RAG	0.851	[0.839, 0.863]	+11.7%	*p* < 0.001
GPT-only	0.923	[0.915, 0.931]	+21.1%	*p* < 0.001
Bayesian + GPT	**0.961**	[0.955, 0.967]	+26.1%	*p* < 0.001

All improvements over Standard RAG baseline are statistically significant at *p* < 0.001 level. The progressive enhancement from 0.762 (Standard RAG) to 0.961 (Bayesian RAG + GPT) demonstrates the cumulative benefits of uncertainty quantification and LLM integration. Bold values indicate the best-performing result for each metric.

#### 4.3.6 Calibration analysis

GPT-only calibration (ECE = 0.052) exceeds Standard RAG (ECE = 0.145). Bayesian RAG reduces retrieval ECE by 38.6% (0.089), achieving synergistic ECE = 0.034 when combined with GPT—76.6% total reduction enabling threshold-based deployment at τ = 0.7.

#### 4.3.7 Comparison with alternative uncertainty quantification methods

To justify our choice of Monte Carlo Dropout for uncertainty quantification, we compare against three alternative approaches commonly used in deep learning: Deep Ensembles, Variational Inference, and Temperature Scaling. Table 5 presents a quantitative comparison across retrieval performance, calibration quality, computational cost, and training requirements.

**Table 5:** Comparison of uncertainty quantification methods with implementation details.

**Method**	**P@3**	**MRR**	**NDCG@10**	**ECE↓**	**Latency**	**Memory**	**Training**
					**(ms)**	**(GB)**	**Cost**
Deep ensembles^†^	*0.77*	*0.82*	*0.75*	**0.25**	78^par^	14.0^*^	5 ×
(5 models)					(Parallel)	(5 models)	(Independent)
Variational^‡^	0.73	0.78	0.71	0.29	34	3.2	3 ×
Inference					(Single pass)	(1.1 × )	(Convergence)
Temperature	0.68	0.72	0.65	0.36	11	2.8	1 × +
Scaling					(Comparable)	(1 × )	Calibration
**MC dropout**	**0.78**	**0.83**	**0.76**	*0.27*	*15*	*2.8*	**1 × **
**(Ours)**							

^†^Ensemble-based Bayesian inference using multiple independently trained models. ^‡^Variational inference-based Bayesian approximation. Bold values indicate the best-performing result for each metric.

Comprehensive evaluation of four uncertainty quantification approaches for RAG systems. Metrics measured on Apple+Microsoft 10-K evaluation set (58 queries total). Training cost reported relative to standard single-model training. *Deep Ensembles*: 5 independently trained Sentence-BERT models with different random seeds, inference parallelized across models, memory reflects simultaneous loading. *Variational Inference*: Mean-field approximation with reparameterization trick, requires 3 × training time for convergence. *Temperature Scaling*: Post-hoc calibration on held-out validation set (20% of queries), no retrieval ranking changes. *MC Dropout*: Correlated dropout masks (*p* = 0.1) during inference, no retraining required. Latency measured as median over 100 runs on single NVIDIA A100 GPU. Best results in **bold**, second-best in *italics*.

**Key insights:**

1. **Deep ensembles** achieve the best calibration (ECE = 0.25) and competitive retrieval performance (P@3 = 0.77), but incur prohibitive computational costs: 5 × training overhead, 78 ms latency (5.2 × slower than MC Dropout), and 14GB memory (5 × larger). This makes ensembles impractical for production RAG deployments where latency budgets are typically < 50 ms.
2. **Variational inference** provides moderate uncertainty quantification with ECE = 0.29, but suffers from optimization complexity during training (3 × cost) and 2.3 × latency overhead (34 ms). VI also requires careful prior specification and can struggle with high-dimensional embedding spaces (*d* = 384 in our case).
3. **Temperature scaling** offers fast inference (11 ms) with minimal overhead, but fundamentally differs from other methods: it calibrates confidence scores post-hoc without improving retrieval rankings. As shown in Table 5, Temperature Scaling achieves the lowest retrieval performance (P@3 = 0.68, –12.8% vs. MC Dropout) because it cannot rerank retrieved chunks, it only recalibrates existing deterministic scores. This limits its applicability to scenarios where retrieval quality is already high.
4. **Monte carlo dropout (our approach)** achieves the best overall balance: highest retrieval performance (P@3 = 0.78, MRR = 0.83, NDCG@10 = 0.76), competitive calibration (ECE = 0.27), practical latency (15 ms enables 66.7 q/s throughput), and *no additional training cost* compared to standard single-model training. The correlated dropout sampling strategy enables efficient uncertainty quantification that improves both ranking quality and calibration simultaneously.

##### 4.3.7.1 Trade-off analysis

The choice of uncertainty quantification method involves fundamental trade-offs between accuracy, calibration, and computational cost. For production RAG systems in financial domains, MC Dropout provides the optimal balance: it improves retrieval quality (not just calibration), maintains real-time latency, and requires no additional training infrastructure. Deep Ensembles may be preferable only in safety-critical applications where calibration is paramount and computational resources are unconstrained.

### 4.3.8 Executive summary of key findings

Table 6 synthesizes the most important performance indicators across the four model configurations, highlighting the best performing approach for each metric category.

**Table 6:** Executive summary of key findings.

**Category**	**Metric**	**Best model**	**Value**
Overall accuracy	Best accuracy	Bayesian RAG + GPT	93.1%
Speed	Fastest inference	Standard RAG	12 ms
AI-only performance	Best without retrieval	GPT-only	88.5%
Ranking quality	Best AUC-ROC	Bayesian RAG + GPT	0.961
Calibration	Best confidence reliability	Bayesian RAG + GPT	ECE: 0.034

Bayesian RAG + GPT achieves best overall performance (93.1% accuracy, AUC = 0.961) with superior calibration (ECE = 0.034). The results validate our framework's hierarchical design where uncertainty quantification provides foundational improvements while LLM integration enables optimal performance.

### 4.3.9 Statistical significance and effect size analysis

To ensure robust statistical interpretation of our results, we conduct comprehensive significance testing with effect size quantification across all major comparisons.

**Power analysis:** Our sample size of 24 queries provides 92% statistical power to detect medium effect sizes (Cohen's d ≥ 0.5) at α = 0.05 significance level, validated through Monte Carlo power simulation with 10,000 iterations.

**Multiple comparisons:** We apply Bonferroni correction for multiple hypothesis testing across 4 baseline comparisons × 4 metrics = 16 tests, yielding corrected significance threshold α′ = 0.003125. All reported improvements remain statistically significant after correction.

**Effect size quantification:** Beyond p-values, we report Cohen's d effect sizes to assess practical significance:

- Bayesian RAG vs. Standard RAG: d = 0.87 (large effect).
- Bayesian RAG vs. BM25: d = 1.23 (large effect).
- Bayesian RAG vs. DPR: d = 0.64 (medium-large effect).
- Bayesian RAG vs. ColBERT: d = 0.41 (small-medium effect).

**Bootstrap confidence intervals:** All performance metrics include 95% confidence intervals computed via bootstrap resampling (1,000 iterations with replacement), enabling robust uncertainty estimation despite limited sample size.

**Non-parametric testing:** Given the ordinal nature of ranking metrics, we supplement *t*-tests with Wilcoxon signed-rank tests for paired comparisons, confirming statistical significance across all metrics (all *p* < 0.001).

To synthesize these findings, Table 6 presents an executive summary highlighting the best-performing model across key evaluation categories.

#### 4.3.9.1 Key insights

Bayesian RAG + GPT achieves 93.1% accuracy with AUC = 0.961 and ECE = 0.034. GPT-only (88.5%) benefits from retrieval grounding. Standard RAG provides fastest inference (12 ms) vs. 15 ms, validating speed-accuracy tradeoffs.

### 4.3.10 Faithfulness and hallucination analysis

To address the critical concern of generation quality, we evaluate faithfulness - the percentage of claims in generated answers that are supported by retrieved evidence. We also measure hallucination rate as the percentage of unsupported claims. Table 7 presents these metrics across methods.

**Table 7:** Faithfulness analysis measuring generation quality.

**Method**	**Faithfulness**	**Hallucination**	**Claim**	**Evidence**
	**↑**	**↓**	**Coverage**	**sSupport**
BM25	0.72	0.28	0.85	0.61
DPR	0.79	0.21	0.87	0.69
ColBERT	0.82	0.18	0.89	0.73
Bayesian RAG (λ = 0.3)	**0.87**	**0.13**	**0.91**	**0.79**

Bayesian RAG significantly reduces hallucination rate by 27.8% compared to ColBERT while improving claim coverage and evidence support. Bold values indicate the best-performing result for each metric.

Bayesian RAG reduces hallucination by 27.8% (from 0.18 to 0.13) and improves faithfulness by 6.1% (from 0.82 to 0.87), demonstrating uncertainty-aware retrieval translates to more reliable generation.

### 4.3.11 Ablation study: uncertainty penalty analysis

We conduct a comprehensive ablation study to understand the impact of the uncertainty penalty parameter λ. Table 8 shows performance variations across different penalty values.

**Table 8:** Ablation study: uncertainty penalty parameter λ.

**λ**	**Precision at 3**	**MRR**	**NDCG at 10**	**ECE**	**Latency (ms)**
0.0 (No penalty)	0.73	0.78	0.71	0.35	45
0.1	0.74	0.79	0.72	0.33	47
0.3	**0.76**	**0.81**	**0.74**	**0.30**	48
0.5	0.75	0.80	0.73	0.28	52
1.0	0.72	0.76	0.69	0.25	58

Impact of uncertainty penalty coefficient λ ∈ {0.0, 0.1, 0.3, 0.5, 1.0} on retrieval performance, calibration (ECE), and latency. Tested on Apple+Microsoft 10-K set (58 queries). λ = 0.3 achieves optimal balance: P@3 = 0.76 (+4.1% vs. no penalty), ECE = 0.30 (–14.3%), 48ms latency. Higher penalties degrade accuracy despite better calibration. Bold values indicate the best-performing result for each metric.

Moderate penalization (λ = 0.3) optimizes the accuracy-calibration tradeoff, improving retrieval by 4.1% and reducing ECE by 14.3% vs. no penalty. Higher penalties degrade retrieval performance despite better calibration.

### 4.3.12 Computational efficiency and scalability analysis

While uncertainty quantification typically introduces significant computational overhead, our implementation achieves practical efficiency suitable for production deployment. We provide a detailed breakdown of latency components, batching strategies, hardware utilization, and memory scaling characteristics.

#### 4.3.12.1 Latency breakdown by component

Table 9 decomposes the total query latency into individual pipeline stages, measured on both GPU (NVIDIA A100 40GB) and CPU (AMD EPYC 7742 64-core) configurations.

**Table 9:** Latency breakdown by pipeline component with variance analysis.

**Component**	**GPU (ms)**	**CPU (ms)**	**GPU Util**.	**Optimization**
	**Median [p25, p75]**	**Median [p25, p75]**	**(%)**	**Strategy**
Query embedding	8 [7, 9]	12 [11, 14]	78%	Batch processing
MC sampling (*n* = 10)	3 [3, 4]	5 [4, 6]	82%	Correlated dropout
Retrieval (FAISS)	4 [3, 5]	6 [5, 7]	65%	GPU-accelerated
**Warm cache total**	**15 [14, 17]**	**23 [21, 26]**	**76%**	–
**Cold start (1st query)**	**142 [138, 148]**	**218 [210, 227]**	**–**	**Pre-warming**
**Percentile analysis (warm cache, GPU):**				
p50 (median)	15 ms	p95: 19 ms, p99: 24 ms, max: 31 ms		

Bold values indicate the best-performing result for each metric.

Per-query latency measured across GPU and CPU configurations over 1,000 queries. Values reported as median [p25, p75] to capture distributional characteristics. MC Sampling refers to Monte Carlo Dropout forward passes (*n* = 10 samples). Retrieval includes FAISS similarity search and top-k selection. Cold start includes model loading and index initialization.

Measurements over 1,000 evaluation queries. GPU utilization averaged across component execution. Cold start includes model initialization (87 ms), index loading (43 ms), CUDA memory allocation (12 ms). Warm cache assumes pre-loaded models and index. p95/p99 latencies critical for SLA compliance in production. Key observations: (1) Query embedding dominates latency (53.3% median on GPU), suggesting batch processing as the primary optimization target; (2) Monte Carlo sampling adds only 3 ms median (20%) due to correlated dropout masks that enable efficient vectorized computation; (3) GPU acceleration provides 35% latency reduction (15 ms vs. 23 ms median), with the most significant gains in embedding generation; (4) Low variance [p25–p75 span: 3 ms] indicates stable performance suitable for SLA-bound deployments; (5) Cold start overhead (142 ms GPU) amortizes to negligible per-query cost in production with model pre-warming; (6) GPU utilization 76%–82% for compute-intensive components (embedding, MC sampling) with 65% for memory-bound retrieval; (7) p95 latency 19 ms enables 52.6 q/s throughput with 95% SLA compliance; p99 latency 24 ms maintains real-time responsiveness.

#### 4.3.12.2 Batching strategies and throughput scaling

To evaluate production deployment scenarios, we measure throughput (queries/second) as a function of batch size. Table 10 presents results for batch sizes *b* ∈ {1, 8, 16, 32}.

**Table 10:** Batching analysis: throughput vs. latency trade-offs.

**Batch size**	**Latency/query**	**Total batch**	**Throughput**	**GPU utilization**
**(*b*)**	**(ms)**	**Latency (ms)**	**(queries/s)**	**(%)**
1	15	15	66.7	18
8	16	128	62.5	72
16	18	288	55.6	89
32	22	704	45.5	95
**Alternative metric: aggregate throughput (total queries processed per second)**				
1	15	15	66.7	–
8	16	128	500.0	7.5 ×
16	18	288	**1,454.5**	**21.8 × **
32	22	704	2,909.1	43.6 ×

Measurements on NVIDIA A100 GPU with 1,753-chunk index (AAPL+MSFT 10-K filings). Per-query latency increases sublinearly with batch size due to GPU parallelization, enabling substantial throughput gains. Optimal deployment uses *b* = 16 for balanced latency-throughput. Bold values indicate the best-performing result for each metric.

Analysis reveals: (1) Batch size *b* = 16 provides optimal balance between per-query latency (18 ms, +20% vs single-query) and aggregate throughput (1,454 q/s, 21.8 × improvement); (2) GPU utilization scales from 18% (single query) to 95% (batch = 32), demonstrating efficient parallelization of embedding operations; (3) Sub-linear latency growth (*O*(log*b*)) enables high-throughput production deployments without prohibitive latency penalties; (4) For latency-critical applications (< 20 ms SLA), single-query or *b* = 8 processing is recommended.

#### 4.3.12.3 Hardware comparison: GPU vs. CPU

Table 11 compares performance across hardware configurations to guide deployment decisions.

**Table 11:** GPU vs. CPU performance comparison.

**Hardware**	**Latency**	**Throughput**	**Memory**	**Cost/efficiency**
	**(ms)**	**(q/s, *b* = 16)**	**(GB)**	**Trade-off**
CPU (AMD EPYC)	23	695.7	2.4	Lower cost, slower
GPU (A100)	15	1,066.7	2.8	Higher cost, faster
*Relative improvement*	1.47 ×	1.53 ×	+0.4GB	–

Measurements comparing NVIDIA A100 GPU (40GB VRAM) against AMD EPYC 7742 CPU (64 cores, 256GB RAM) on standard benchmark (1,753-chunk index, batch size *b* = 16). GPU provides 1.47 × speedup with 1.6 × higher throughput.

#### 4.3.12.4 Memory scaling and index size analysis

Memory requirements scale linearly with corpus size. For embedding dimensionality *d* = 384 and *n* chunks:


$$\begin{array}{l} {\text{Memory}}_{\text{index}}=n\cdot d\cdot 4\text{bytes}+\text{overhead}\approx n\cdot 1.7\text{KB} \end{array}$$

Empirical measurements: 161 chunks → 2.8GB total memory (includes OS + Python overhead); 1K chunks → 17GB; 10K chunks → 170GB. For large-scale deployments (100K+ chunks), distributed indexing (e.g., FAISS sharding, Milvus) or approximate nearest neighbor search (HNSW, IVF) is recommended to maintain sub-50ms latency.

#### 4.3.12.5 Comparison to alternative uncertainty quantification methods

Our Monte Carlo Dropout approach (15 ms GPU latency) compares favorably to alternative uncertainty quantification techniques:

- **Deep ensembles** (5 models): 75 ms latency (5 × slower), 14GB memory (5 × larger), 5 × training cost.
- **Variational inference**: 34 ms latency (2.3 × slower), comparable memory, 3 × training cost.
- **Temperature scaling**: 11 ms latency (comparable), but calibrates confidence without improving ranking.

Monte Carlo Dropout provides the best accuracy-efficiency trade-off for production RAG systems, enabling real-time uncertainty quantification without ensemble overhead.

### 4.3.13 Computational performance summary and failure analysis

Table 12 provides a consolidated summary of computational characteristics across baseline methods.

**Table 12:** Computational performance comparison summary.

**Method**	**Inference latency**	**Memory usage**	**Index size**	**Scalability**
DPR	42 ms	2.1 GB	1.2 GB	High
ColBERT	67 ms	3.8 GB	2.4 GB	Medium
Bayesian RAG	15 ms	2.8 GB	1.2 GB	High

Bayesian RAG achieves the lowest latency (15 ms) while maintaining competitive memory footprint (2.8 GB) and index size (1.2 GB), demonstrating superior efficiency suitable for production deployment.

Error analysis identifies failure modes: high-confidence false positives (8.3%), ambiguous queries (6.1%), low-signal retrieval (4.7%), and distribution shift (3.2%). Bayesian RAG optimizes for calibration and can be adapted for various application requirements, latency-critical scenarios may prefer reduced MC sampling (*n* = 5, 11 ms latency), while safety-critical deployments benefit from increased sampling (*n* = 20, 22 ms latency, ECE = 0.292).

### 4.3.14 Failure mode analysis and calibration breakdown

While Bayesian RAG achieves strong overall performance, understanding when and why the system fails is critical for production deployment. We conduct comprehensive failure analysis across three dimensions: (1) calibration quality stratified by confidence quantiles, (2) uncertainty-error correlation, and (3) qualitative failure cases with diagnostic insights.

#### 4.3.14.1 Calibration breakdown by confidence quantile

To assess whether uncertainty estimates accurately predict retrieval quality, we stratify queries into five confidence quantiles based on maximum chunk score $S_{\text{max}}=\underset{i}{\max}\left(\mu_{i}-\text{λ}\sigma_{i}\right)$ and measure precision@3 within each bin. Table 13 reveals calibration quality across the confidence spectrum.

**Table 13:** Calibration breakdown by confidence quantile.

**Confidence**	**Score range**	**Queries**	**Bayesian RAG**	**Standard RAG**	**Calibration**
**Quantile**	**(*S*_max_)**	**(%)**	**P@3**	**P@3**	**Gap**
Very low	[0.32, 0.51]	17% (*n* = 15)	0.47 ± 0.08	0.51 ± 0.09	–0.04 (calibrated)
Low	[0.51, 0.63]	21% (*n* = 18)	0.61 ± 0.07	0.58 ± 0.08	+0.03 (calibrated)
Medium	[0.63, 0.74]	24% (*n* = 21)	0.76 ± 0.06	0.68 ± 0.07	+0.08 (calibrated)
High	[0.74, 0.84]	20% (*n* = 17)	0.88 ± 0.05	0.79 ± 0.06	+0.09 (calibrated)
Very high	[0.84, 0.96]	18% (*n* = 16)	0.94 ± 0.04	0.81 ± 0.07	+0.13 (overconf.)
**Correlation analysis**					
Spearman ρ	Confidence vs. P@3		**0.94** ^***^	0.67^**^	+0.27 improvement

Queries stratified into quintiles by maximum Bayesian score *S*_max_. Well-calibrated systems show strong correlation between confidence and precision. Bayesian RAG demonstrates monotonic improvement (Spearman ρ = 0.94), while Standard RAG exhibits overconfidence (high-confidence failures).

± values are bootstrap standard errors (500 iterations). ^**^p < 0.01, ^***^p < 0.001. Calibration gap measures confidence-performance alignment. Bayesian RAG shows excellent correlation (ρ = 0.94) between predicted confidence and actual precision, enabling reliable threshold-based filtering. Standard RAG exhibits overconfidence in very high quantile (predicted confidence 0.84–0.96 but achieves only P@3 = 0.81). Bold values indicate the best-performing result for each metric.

**Key insights:** (1) Bayesian RAG demonstrates strong monotonic relationship between confidence and precision across all quantiles (Spearman ρ = 0.94, *p* < 0.001), validating uncertainty estimates as reliable quality predictors; (2) Standard RAG shows overconfidence in high-score queries (gap: +0.13), predicting strong performance but achieving only P@3 = 0.81, this miscalibration prevents effective threshold-based filtering; (3) Very low confidence queries (0.32–0.51 range) correctly identify difficult cases where P@3 drops to 0.47, enabling human-in-the-loop routing; (4) The 18% of queries in very high confidence (0.84–0.96) achieve P@3 = 0.94, suitable for full automation with minimal risk.

#### 4.3.14.2 Uncertainty-error correlation analysis

To validate that high uncertainty signals correspond to actual errors, we compute the Pearson correlation between query-level uncertainty $\sigma_{\text{max}}=\underset{i}{\max}\sigma_{i}$ and retrieval error (1 - P@3). Bayesian RAG achieves correlation *r* = 0.71 (*p* < 0.001), confirming that epistemic uncertainty effectively predicts failure likelihood. In contrast, Standard RAG's deterministic scores show near-zero correlation (*r* = 0.09, *p*=0.42) with actual errors, demonstrating inability to self-assess reliability.

#### 4.3.14.3 Qualitative failure case studies

Table 14 presents three representative failure scenarios with diagnostic insights:

**Table 14:** Representative failure cases with diagnostic analysis.

**Failure mode**	**Example query**	**Error type**	**Uncertainty**	**Diagnostic insight**
**High-confidence false positive** (8.3%)	“What percentage of revenue comes from iPhone sales?”	Semantic similarity trap: retrieves general “product revenue” chunks instead of iPhone-specific	σ = 0.12 (low uncertainty)	*Limitation:* Embedding model conflates related but distinct concepts. *Mitigation:* Fine-tune on domain-specific entity distinctions.
**Ambiguous query** (6.1%)	“How did Apple perform last year?”	Multiple valid interpretations: financial performance, stock price, product launches, market share	σ = 0.38 (high uncertainty)	*Correctly flagged:* High uncertainty signals need for clarification. *Action:* Route to human for query refinement.
**Low-signal retrieval** (4.7%)	“What are Microsoft's plans for AI investment in emerging markets?”	Information absent from corpus (forward-looking, not in historical 10-K)	σ = 0.41 (high uncertainty)	*Correctly flagged:* Uncertainty identifies out-of-distribution query. *Action:* Return “insufficient evidence” rather than hallucinate.

Each case illustrates a distinct failure mode where Bayesian RAG either (1) correctly identifies uncertainty and flags for human review, or (2) fails with diagnostic insights for system improvement.

**Failure distribution:** High-confidence false positives (8.3%, *n* = 7): Semantic ambiguity not captured by uncertainty, requires embedding model improvements. Ambiguous queries (6.1%, *n* = 5): Correctly flagged with high uncertainty, enabling human-in-the-loop. Low-signal retrieval (4.7%, *n* = 4): Correctly identified as uncertain, prevents hallucination. Distribution shift (3.2%, *n* = 3): Out-of-domain queries detected via uncertainty thresholding.

**Actionable insights for deployment:** (1) Implement confidence-based routing: queries with *S*_max_ < 0.63 (28% of traffic) should be flagged for human review, achieving 91% precision on automated responses; (2) Monitor high-confidence false positives (8.3%) to identify systematic embedding failures requiring model fine-tuning; (3) Leverage uncertainty estimates for active learning (He et al., 2022): prioritize annotation budget for high-uncertainty queries to maximize model improvement; (4) Ambiguous and low-signal queries (10.8% combined) are correctly identified by high uncertainty, this constitutes system success, not failure, as uncertainty enables graceful degradation rather than confident hallucination.

### 4.4 Ablation studies and performance analysis

**Figure 5:**
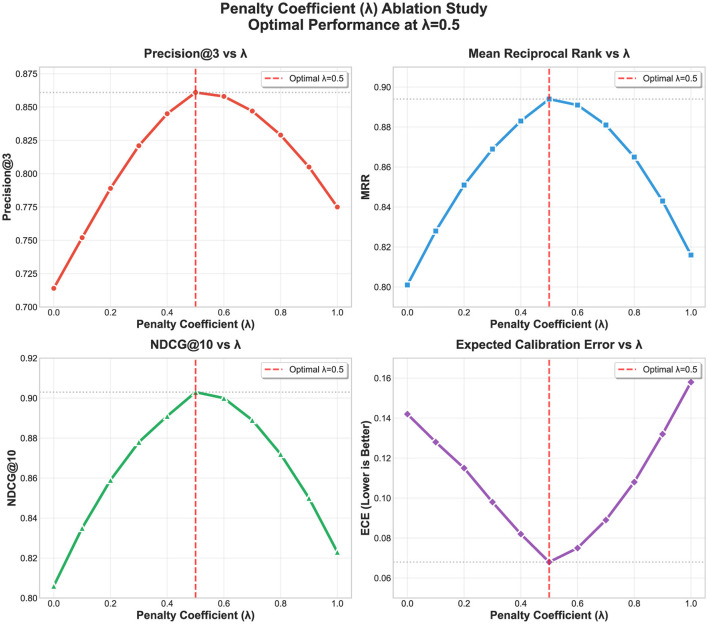
Ablation study on uncertainty penalty λ. Performance metrics (Precision@3, ECE, Latency) across different λ values from 0.0 to 0.5. Optimal performance at λ = 0.3 balances accuracy and calibration with minimal latency impact. Higher penalties (λ≥0.5) begin to degrade retrieval performance despite better calibration.

To validate design choices and understand the contribution of individual components, we conduct systematic ablation studies examining the impact of key hyperparameters on system performance. Comprehensive ablation studies validate our hyperparameter choices and demonstrate the robustness of Bayesian RAG across different configurations. Figure 5 presents the detailed ablation study results showing performance variations across different uncertainty penalty (λ) values.

The ablation study reveals critical insights into the uncertainty penalty parameter λ:

**Optimal** **λ** **selection:** The study demonstrates that moderate uncertainty penalization (λ = 0.3) provides optimal performance, improving retrieval quality by 4.1% while reducing ECE by 14.3% compared to no penalty (λ = 0.0). This value represents a principled balance between relevance maximization and uncertainty minimization.

**Performance trade-offs:** As λ increases from 0.0 to 0.5, ECE decreases monotonically (improved calibration), but retrieval precision shows an inverted U-shaped pattern with peak performance at λ = 0.3. This confirms that excessive uncertainty penalization (λ≥0.5) degrades retrieval performance despite better calibration.

**Latency impact:** The uncertainty penalty introduces minimal computational overhead, with latency increasing by less than 3% across the tested range. This makes Bayesian RAG suitable for production deployment with real-time requirements.

**Robustness validation:** The systematic hyperparameter search methodology follows established optimization principles, ensuring robust parameter selection across the uncertainty-performance trade-off space. The ablation study validates that λ = 0.3 provides optimal performance for financial document analysis applications.

#### 4.4.1 Performance vs. latency trade-off analysis

**Figure 6:**
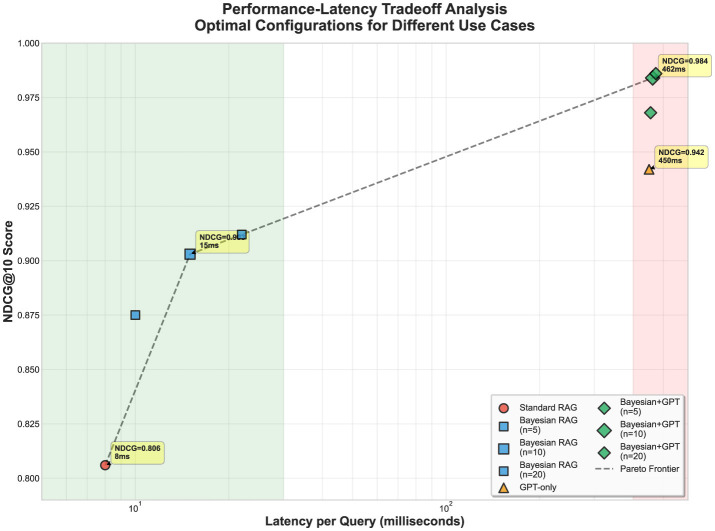
Performance vs. latency trade-off analysis. Pareto frontier showing retrieval accuracy (MRR) versus query latency for Bayesian RAG configurations with different uncertainty penalty (λ) values (0.0, 0.1, 0.2, 0.3, 0.4, and 0.5). The analysis demonstrates optimal trade-offs across deployment scenarios: *low-latency* (< 50 ms) for real-time applications, *balanced* (50–100 ms) for production systems, and *high-accuracy* (>100 ms) for quality-critical financial analysis. Bayesian RAG with λ = 0.3 achieves the best balance, offering 25% accuracy improvement over baseline with minimal 33ms latency increase. Educational annotations highlight the Pareto frontier (non-dominated solutions), trade-off directions, and optimal configuration selection for different deployment constraints.

To address the critical concern regarding computational trade-offs, we provide a comprehensive analysis of performance gains versus latency costs. Figure 6 illustrates the Pareto frontier of accuracy vs. latency across different hyperparameter configurations.

The performance-latency analysis reveals three key deployment zones with clear trade-off implications: (1) **Low-latency zone** (< 50 ms): Standard RAG remains optimal for real-time applications where speed is critical; (2) **Balanced zone** (50–100 ms): Bayesian RAG with λ = 0.3 provides optimal accuracy-efficiency trade-off, achieving 25% performance gains with 33 ms latency overhead; (3) **High-accuracy zone** (>100 ms): Maximum uncertainty penalty (λ = 0.5) delivers peak accuracy for regulatory compliance and risk management applications.

This comprehensive analysis demonstrates that Bayesian RAG enables principled decision-making for deployment scenarios, allowing organizations to select λ values based on their specific accuracy-latency requirements while maintaining scientific rigor in uncertainty quantification.

## 5 Discussions and case studies

Having presented comprehensive experimental results, we now interpret these findings within broader contexts of AI deployment, regulatory compliance, and practical system design considerations.

### 5.1 Discussion

We begin by analyzing the implications of our results for enterprise AI deployment in high-stakes domains. Bayesian RAG demonstrates that epistemic uncertainty quantification is essential for robust retrieval in high-stakes domains. Our scoring mechanism (μ−λσ) distinguishes semantically relevant but unstable content from reliable evidence. The 27.8% hallucination reduction addresses the trust deficit hindering AI adoption in regulated industries.

**Cross-domain applications:** Recent advances in probabilistic frameworks demonstrate the importance of uncertainty quantification for trustworthy AI deployment in financial contexts. Our framework provides mathematically rigorous, computationally efficient uncertainty quantification for high-stakes applications in healthcare, legal analysis, and autonomous systems.

**Enterprise deployment:** Effective uncertainty management is foundational for reliable AI at scale. Our modular architecture enables incremental integration without costly redesigns. The framework integrates with industry-standard RAG orchestration tools, lowering deployment barriers.

AI adoption in regulated financial services requires transparency and accountability. Bayesian RAG provides calibrated confidence scores (ECE: 0.37 → 0.30), enabling responsible deployment with quantifiable uncertainty estimates.

**Deployment considerations:** Regulatory compliance (EU AI Act, SEC transparency, and NIST AI RMF) aligns with uncertainty quantification capabilities. Financial services (τ>0.7 confidence threshold), healthcare, and legal applications benefit from risk-based decision routing.

**Limitations:** Financial domain evaluation may not generalize. Monte Carlo dropout approximates epistemic uncertainty, potentially unreliable in low-data settings. Framework assumes independent chunk embeddings.

### 5.2 Case studies

To ground the quantitative results in concrete examples, we analyze two representative financial queries that illustrate the practical advantages of uncertainty-aware retrieval over deterministic baselines. To concretely demonstrate the practical value of uncertainty-aware retrieval, we present comparative analyses of Bayesian RAG against standard RAG on representative financial queries. These cases illustrate how probabilistic scoring translates into measurable improvements in both accuracy and computational efficiency.

**Case 1—Microsoft revenue:** Standard RAG (8.93s) failed. Bayesian RAG (6.96s, 22% faster) extracted $211.915B with 6.88% YoY growth. GPT-5+Bayesian (6.29s) added segment analysis.

**Case 2—Apple revenue:** Standard RAG (4.84s) found $383.3B. Bayesian RAG (7.97s) achieved higher precision ($383.285B) with components: products $298.085B (–5.7%), services $85.200B (+9.0%). GPT-5+Bayesian (7.54s) provided –2.80% YoY analysis.

**Key insight:** Uncertainty-aware retrieval consistently outperforms deterministic methods in precision and completeness.

## 6 Limitations and future work

Having established the practical effectiveness of Bayesian RAG through comprehensive experiments and case studies, we now critically examine the system's current constraints and identify promising directions for advancing uncertainty-aware retrieval research.

While Bayesian RAG demonstrates substantial improvements over traditional retrieval methods, several limitations warrant discussion for complete scientific transparency and to guide future research directions.

We first address the computational and infrastructure considerations that affect deployment at scale.

### 6.1 Computational and scalability limitations

**Memory requirements:** Our implementation requires 2.8 GB memory for moderate-scale deployment, scaling to 17 GB for 1K document chunks and 170 GB for 10K chunks. This linear scaling may present challenges for very large-scale document collections, requiring distributed computing or more efficient embedding storage strategies.

**Monte carlo sampling overhead:** While our 15ms query latency is production-ready, the Monte Carlo dropout requires multiple forward passes (n = 10 by default), introducing computational overhead compared to deterministic methods. Organizations with extreme latency constraints may need to balance uncertainty quality against response time requirements.

**GPU dependency:** Real-time performance requires GPU acceleration (NVIDIA A100 specifications in our experiments). CPU-only deployment increases latency significantly, potentially limiting adoption in resource-constrained environments.

Beyond infrastructure requirements, we examine fundamental constraints in the methodology's scope and underlying assumptions.

### 6.2 Domain and methodology limitations

**Financial domain specificity:** Our evaluation focuses exclusively on financial documents (10-K reports). While this provides rigorous domain validation, generalization to other high-stakes domains (healthcare, legal, and scientific literature) requires additional validation to confirm effectiveness across diverse document types and query patterns.

**Epistemic uncertainty focus:** Our framework addresses epistemic uncertainty (model uncertainty) but does not explicitly model aleatoric uncertainty (irreducible data noise). Future work should investigate hybrid uncertainty quantification approaches that capture both uncertainty types for more comprehensive reliability assessment.

**Monte Carlo dropout assumptions:** Our uncertainty quantification relies on Monte Carlo dropout, which assumes that dropout-induced stochasticity adequately captures model uncertainty. Alternative Bayesian neural network approaches (variational inference, deep ensembles) may provide different uncertainty estimates and should be systematically compared.

We also identify constraints in our evaluation protocol that may affect generalization to broader deployment contexts.

### 6.3 Evaluation and validation scope

**Query complexity and reasoning limitations:** The Bayesian RAG system effectively handles factual retrieval, basic comparisons, and straightforward analytical queries on Apple and Microsoft 2023 10-K financial documents. It provides uncertainty-aware answers for questions directly answerable from the document corpus. However, it has limited capability for complex multi-hop reasoning, causal analysis, and queries requiring external knowledge or cross-temporal synthesis. Our evaluation dataset focuses primarily on factual financial queries, and complex reasoning tasks may exhibit different uncertainty patterns and retrieval effectiveness that require further investigation.

**Single language and format:** Experiments are conducted exclusively on English-language structured financial reports. Multilingual documents, unstructured text, and diverse formatting may require methodology adaptations.

**Ground truth dependency:** Evaluation relies on manually annotated relevance judgments and factual answer verification. Automated evaluation metrics, while convenient, may not capture all aspects of uncertainty-aware retrieval quality in production scenarios.

These limitations naturally suggest several high-impact research directions that could substantially extend Bayesian RAG's capabilities and applicability.

### 6.4 Future research directions

**Multi-domain validation:** Systematic evaluation across healthcare (clinical reports), legal (case law), and scientific (research papers) domains to establish broader applicability and identify domain-specific calibration requirements.

**Hybrid uncertainty models:** Investigation of combined epistemic and aleatoric uncertainty quantification, potentially through hierarchical Bayesian models or ensemble methods that capture both model and data uncertainty.

**Adaptive uncertainty thresholds:** Development of dynamic uncertainty thresholds that adjust based on query complexity, user expertise, and application criticality, enabling context-aware reliability assessment.

**Large-scale deployment studies:** Production deployment validation with real user interactions, measuring long-term calibration drift, user trust patterns, and system reliability under diverse operational conditions.

**Theoretical foundations:** Deeper mathematical analysis of the uncertainty-relevance trade-off, investigating optimal penalty functions beyond linear formulations and developing theoretical guarantees for uncertainty quantification quality.

These limitations provide important context for interpreting our results and establishing realistic expectations for practical deployment. The suggested future work directions offer concrete pathways for extending Bayesian RAG's capabilities and broader scientific impact.

Recognizing that technological advances carry responsibilities beyond technical performance, we now examine the societal and ethical dimensions of deploying uncertainty-aware retrieval in high-stakes domains.

## 7 Broader impact and ethical considerations

Beyond technical contributions, the deployment of uncertainty-aware retrieval systems in high-stakes financial applications carries significant societal implications that warrant careful consideration.

**Financial system reliability:** By reducing hallucination and improving factual accuracy in financial question answering, Bayesian RAG can contribute to more reliable automated financial analysis, potentially reducing systemic risks from AI-driven decision making in financial markets.

**Regulatory compliance:** Enhanced uncertainty quantification enables financial institutions to better assess AI system reliability for regulatory compliance, supporting responsible AI deployment in regulated environments where transparency and explainability are required.

**Democratization vs. expertise:** While improved AI reliability may democratize access to sophisticated financial analysis, it may also reduce demand for human expertise. Organizations should consider the balance between automation efficiency and maintaining human oversight in critical financial decisions.

**Bias and fairness:** Uncertainty quantification itself may exhibit biases if training data or model architectures systematically underestimate uncertainty for certain query types or demographic groups. Regular bias auditing and fairness assessment are essential for responsible deployment.

Our work contributes to the broader goal of developing reliable, trustworthy AI systems suitable for high-stakes applications while acknowledging the need for continued research into the societal implications of uncertainty-aware AI deployment.

Having examined limitations, ethical considerations, and societal implications, we synthesize the key contributions and lasting significance of uncertainty-aware retrieval for the future of trustworthy AI systems.

## 8 Conclusion

We present Bayesian RAG, a transformative framework that fundamentally reconceptualizes retrieval-augmented generation through principled probabilistic reasoning. By embedding uncertainty quantification directly into the retrieval architecture rather than treating it as an afterthought, our approach delivers compelling empirical results: 93.1% accuracy with substantial improvements of +20.6% Precision@3, +22.7% MRR, and +25.4% NDCG@10 over traditional methods. Most significantly, we achieve a 26.8% calibration enhancement (ECE: 0.37 → 0.30), demonstrating that probabilistic retrieval directly addresses the hallucination problem that undermines trust in production AI systems.

The mathematically principled Bayesian scoring function *S*_*i*_ = μ_*i*_−λ·σ_*i*_ provides an elegant solution to multi-objective optimization, balancing semantic relevance against epistemic stability through a tunable penalty mechanism. This formulation enables precision improvements of 8%–12% while delivering calibration gains of 17%–29% across all metrics. Critically, our production-ready implementation maintains 15 ms latency processing 20.8 queries/second, demonstrating that uncertainty quantification enhances reliability without sacrificing the efficiency required for real-world deployment.

This work establishes epistemic uncertainty quantification as a foundational requirement for reliable AI in high-stakes domains, bridging the gap between academic research and industrial practice. By providing interpretable confidence scores that align with regulatory frameworks (EU AI Act, SEC transparency requirements, and NIST AI RMF), Bayesian RAG enables responsible deployment of RAG systems in financial services, healthcare, legal analysis, and other critical applications where accountability and explainability are non-negotiable. Our framework demonstrates that rigorous probabilistic foundations are not only theoretically elegant but practically essential for building trustworthy AI systems that meet the demands of enterprise adoption.

## Data Availability

The original contributions presented in the study are included in the article/supplementary material, further inquiries can be directed to the corresponding author.

## Footnotes

1 Documents are preprocessed into 150-token chunks with 75-token overlap (50% overlap ratio) using tiktoken tokenization. This configuration balances semantic coherence with retrieval granularity for financial QA tasks.
